# Exploring the determinants on continuance participation of college students toward blended learning: the stimulating role of course characteristics and instructor support

**DOI:** 10.3389/fpsyg.2025.1522810

**Published:** 2025-04-11

**Authors:** Maoyan She, Yiyang Xu, Zhigang Li, Die Hu

**Affiliations:** ^1^College of Management Science, Chengdu University of Technology, Chengdu, Sichuan, China; ^2^Business School of Sichuan University, Sichuan University, Chengdu, Sichuan, China

**Keywords:** course characteristics, instructor support, continuance behavior of blended learning, SOR framework, college students

## Abstract

**Background:**

Blended learning (BL) has become an important learning method in the high education with the rapid advancement of “Internet + Education,” however, college students face notable challenges, such as high dropout rates, low participation and low persistence, which largely reduce the learning effect of BL. Therefore, it is necessary to deeply analyze the question: “What factors will influence college students’ continuance behavior in blended learning (CBBL)?”

**Methods:**

Based on the stimulus-organism-response (SOR) framework and social cognitive theory, this study constructs an integrated model of “*Contextual facilitators–Individual characteristics–Continuance behavior*,” to examine the relationships among the blended course characteristics (BCC), instructor support (IST), individual attributes, such as learning motivation (LM), self-efficacy (SEF) and learning engagement (LET), and college students’ CBBL. Colleting 466 College students who participated in BL through Chinese university MOOCs, the structural equation modeling (SEM) approach was used to test the proposed hypotheses.

**Results:**

The empirical results indicating that, (1) this integrated model explains 62.85% of the variance in college students’ CBBL, and LM, SEF, and LET emerge as the key determinants influencing college students’ CBBL. (2) BCC positively affects LM and LET but has no significant on SEF, and it promotes college students’ CBBL through LM and LET rather than SEF. (3) ITS exerts a significant influence on LM, SEF, and LET, with the most pronounced impact on LET. Moreover, ITS significantly facilitates college students’ CBBL via LM, SEF, and LET.

**Originality/value:**

This study theoretically contributes to literature on BL and extends the application scope of SOR framework. Also, it reveals the antecedents of college students’ CBBL in the BL environment, which is crucial for guiding their continuance learning and promoting the sustainable development of BL education.

## 1 Introduction

The proposal and practice of “Internet + Education” have dramatically promoted educational and instructional reforms, with changes in learning methods, particularly those represented by online learning, being especially evident (Qin and Fang, 2017). As a product of internet development, online learning has greatly facilitated the widespread sharing of high-quality learning resources, overcoming the time and space constraints of traditional learning methods (Dumford and Miller, 2018). However, with the practical development of online learning, issues such as difficulty in achieving effective interaction, learners’ poor self-discipline, and unsatisfactory learning outcomes have also emerged (Yuhanna et al., 2020). Based on this, blended learning (BL), which aims to overcome the drawbacks of both traditional and online learning methods while combining their advantages, has gradually become popular in the field of the high education. For instance, The New Media Consortium Horizon Report in American (Higher Education Edition) listed BL as a rapidly growing trend in the short term for three consecutive years from 2015 to 2017 (Adams Becker et al., 2017). China is also actively promoting the “Double Ten Thousand Plan,” aiming to develop around 10,000 national and provincial top-tier undergraduate courses over 3 years from 2019 to 2021, which includes approximately 4,000 national-level online courses and 6,000 blended top-tier courses, simultaneously, it is also establishing several provincial and university-level online and BL courses (Lv and Li, 2024). The outbreak of COVID-19 and the rapid development of digitalization have greatly accelerated the development of BL. Currently, BL has become one of the most the important learning model for university students to expand learning opportunities, cultivate self-directed learning skills, enhance collaboration abilities, and foster the innovation and critical thinking (Nikolopoulou and Zacharis, 2023). However, in the BL environment, the temporal and spatial disconnection between teaching and learning has led to some students’ reluctance to fully engage, thereby causing issues such as low persistence and high dropout rates (Jiang and Liang, 2023), which significantly undermines the education quality and effectiveness of BL. Therefore, how to ensure college students’ continuance in BL has emerged as a critical issue.

Due to the significance of BL in the high education, an increasing number of literature has paid attention on BL and its determinants. These studies have primarily investigated the factors affecting the BL’s acceptance (Alhramelah and Alshahrani, 2020; Olabisi et al., 2017), satisfaction (Dinh et al., 2021), adaptability (Yang and Pu, 2022), learning effectiveness (Lv and Li, 2024) and the use behavioral intention (Chen et al., 2022; Yang et al., 2023). However, prior studies have predominantly overlooked the continuance behaviors of college students in the BL environment. In the BL process, the effectiveness and success of BL relies heavily on learners’ initiative and self-driven abilities, thus learners must not only continue to form a complete knowledge system by integrating resources both inside and outside the classroom, but also maintain persistent learning behavior to knowledge updating and skill development, so as to fully leverage the advantages of BL model. Therefore, continuance learning behavior is a prerequisite for ensuring the effectiveness of BL. However, the majority of existing studies have investigated the factors influencing learners’ intention of continuance use of BL through the qualitative (Jiang and Liang, 2023; Yu et al., 2024) and the quantitative methods (Baranova et al., 2022; Chen et al., 2022; Yang et al., 2023). Among these studies, most scholars suggested that, internal factors, such as performance expectancy, intrinsic motivation (Yang et al., 2023) and SEF (Jiang and Liang, 2023), individual characteristics, such as attitude and subjective norms (Baranova et al., 2022), perceived usefulness and task-technology fit (Yu et al., 2024), are the key driving force influencing students’ intention to continue learning in a BL environment. But some studies found that academic SEF does not directly impact college students’ continuance intention with BL (Yang et al., 2023). Additionally, external factors, such as course quality and technical support (Chen et al., 2022), teacher’s teaching and SPOC-platform assurance (Jiang and Liang, 2023), social influence and satisfaction (Yu et al., 2024) also determine students’ continuous intention to adopt BL. However, the determinants of college students’ continuance behavior in the BL environment have yet been fully explored (Zacharis and Nikolopoulou, 2022). It is important because it is not aligned between behavioral intention and actual behavior sometime (Venkatesh et al., 2012). Namely, behavioral intention does not fully determine the final actual behavior, which may be influenced by various external and internal factors over time (Limayem et al., 2007; Lin et al., 2005). Moreover, few studies have applied SOR framework in the research field of BL education.

Therefore, this article aims to investigate the determinants of college students’ CBBL through the Chinese university MOOCs platform. The implementation of BL is a comprehensive subject that involves numerous influencing factors, such as course characteristics, external supports and the intrinsic psychological state of students (Jiang and Liang, 2023; Wei et al., 2022), thus college students’ continuance behavior of BL may be affected by these factors simultaneously. Firstly, compared with online learning or offline face-to-face learning courses, the BL courses have new characteristics, such as online learning at students’ own pace and schedule, learning resources integrate various formats, enabling interaction and collaboration with teachers and peers, providing personalized learning contents, and formative and summative evaluations. These course characteristics offer students with flexible, diverse, and personalized learning experiences, which in turn significantly influence their learning state. Secondly, teachers play a pivotal role in the BL environment, taking the responsibility of designing courses that integrate various modes based on the characteristics of the curriculum and the backgrounds of learners (Chen et al., 2022). In the process of BL, if teachers can provide some prosocial instructional behaviors and practices, such as offering diverse learning resources, correcting misunderstandings, guiding in online learning tools and completing assignments, clarifying learning goals, responding promptly and delivering precise feedback, as well as providing constructive recommendations on performance (Feng et al., 2023; Yen and Lee, 2011), college students may be more likely to continually adopt BL. Finally, BL emphasizes the educational philosophy of “student-centered.” College students, as the actual executors of BL, significantly influence continuance behavior through their intrinsic psychological state (Halverson and Graham, 2019; Rafiola et al., 2020), such as motivation, confidence and engagement. However, to the best of our knowledge, few studies have systematically examined the factors influencing college students’ CBBL from both teachers’ and learner’s perspective together. Moreover, the Chinese university MOOCs offers a wealth of online resources, including videos, quizzes, discussion forums, etc. These resources provide opportunities for college students to learn through a BL approach, and also offer teachers with more teaching digital tools. To date, the MOOCs offered by Chinese universities provide access via both websites and mobile applications, which have become a crucial means for students to engage in BL. Nevertheless, a few scholars have studied CBBL among college students through the Chinese university MOOCs (Maes et al., 2023).

Building on the literature regarding BL and its determinants, this study employs the SOR framework to construct a research model *“Contextual facilitators–Individual characteristics–Continuance behavior,”* to exploring the factors influencing college students’ CBBL. Specifically, stimulus refer to various factors in the external environment that can influence learners’ behavior. In the BL courses, students can enhance their learning outcomes by utilizing the advantages of both face-to-face learning and online learning. Meanwhile, instructor support will also influence their state to deal with challenges in the process of BL. Therefore, this stimulation should be considered from both the characteristics of the BL courses and the supports provided by instructors. According to the SOR framework, the individual’s internal state plays a critical mediating role in the influence of stimuli on user’s behavior (Yang J. et al., 2021). Social cognitive theory believes that an individual’s behavior is not only influenced by external environment, but also regulated by psychological and cognitive states (Stajkovic and Luthans, 1998). Students, as active learners, their psychological and cognitive states are able to control the learning behaviors and outcomes, thus factors such as LM and SEF may significantly impact their learning behavior. Additionally, according to Sun et al. (2019), LET, as a positive and continuous emotional state that learners exhibit throughout the duration of learning activities, is also can significantly affect students’ learning behavior. Therefore, this study regards LM, SEF, and LET as the internal behaviors of the organism. Finally, college students’ CBBL is considered as an essential behavioral response.

This study conducts a comprehensive analysis of the factors affecting college students’ CBBL and nine hypotheses are proposed. To empirically test our predictions, we utilize a sample of undergraduate students who have participated in BL through the Chinese university MOOCs, collecting 466 valid questionnaires via the sojump platform. The results obtained from applying the SEM technique with AMOS software indicate that the most proposed hypotheses are supported. This study has the following contributions. On one hand, this paper develops a conceptual model of *“Contextual facilitators–Individual characteristics–Continuance behavior”* to explore the determinants of college students’ CBBL by considering external stimuli and internal individual’ characteristics under the SOR analysis framework, wherein *“S”* represents external contextual facilitators–including BCC and ITS, *“O”* indicates the intrinsic psychological state of students–including LM, SEF, and LET, *“R”* denotes college students’ CBBL, and further deeply analyzes the influencing mechanism and the causal relationships between these variables, thus extending this stream of literature on BL (Lv and Li, 2024; Rafiola et al., 2020; Sun et al., 2017; Yeou, 2016) and the literature adopting the SOR framework as theoretical foundation (He et al., 2022; Yang J. et al., 2021; Zhou and Fang, 2024b). On the other hand, this study holds significant practical implications for improving college students’ intrinsic psychological states and guiding their continuance behaviors in the BL environment. This not only strengthens the practice of lifelong learning among college students but also contributes to the sustainable development and reform of the high education. Additionally, it offers policy recommendations for the construction of BL courses and teachers’ teaching improvement according to the internal and external determinants that affect college students’ CBBL.

## 2 Literature review

### 2.1 BL and its determinants

BL initially emerged from corporate training in foreign enterprises, and has evolved since the late 1990s to the present. BL combines online learning with offline classroom instruction, which has become an innovative and flexible teaching and learning model in the field of education (Ho et al., 2023). As for the definition of BL, scholars have put forward their own understanding from different perspectives. For instance, Garrison and Kanuka (2004) defined BL as the intentional integration of in-classroom face-to-face instruction with online learning experiences. Graham (2006) suggested that BL systems integrate traditional face-to-face instruction with machine learning technologies. Wasoh (2016) considered that BL represents an educational framework that integrates mobile communication devices, web-based learning environments as well as in-person classroom discussions. In the context of “Internet + Education,” the rapid development of emerging technologies such as big data, cloud computing and artificial intelligence has significantly accelerated the application of BL (Yu et al., 2024). The effective implementation of BL also extends beyond simply merging technology with teaching strategies, and it focuses on enhancing highly participation and providing personalized learning experiences that place learners at the center. Therefore, scholars have also suggested that BL is “centered on the student learning experience” (Goodyear and Casey, 2015) or “student-centered learning approach” (Yamin and Ishak, 2017). So far, existing studies have reached a consensus that BL extends beyond its basic definition by integrating a range of elements, such as learning theories, technological tools, mixed learning environments, flexible schedules, diverse assessments and various teaching strategies (Broadbent et al., 2021; Lv and Li, 2024; Zydney et al., 2020). The aim of this holistic integration is reducing costs, maximizing teaching resources, promoting deep learning, and finally improving overall learning effectiveness (Maes et al., 2023). Moreover, the advantages of BL have been widely identified, among which are effective use of time, easier access to the teaching materials, faster and instantaneous communication, greater diversity of materials available, the more flexibility of learning time and space, as well as the better development of students’ self-control, regulation, independently learning ability (Celestino and Noronha, 2021; Zhao, 2022).

A substantial number of studies have investigated the factors influencing BL. In earlier studies, the determinants in driving initial adoption of BL have been explored. These studies primarily integrated the technology acceptance model (TAM) or the unified theory of acceptance and use of technology (UTAUT) with other variables or models to investigate the initial learners’ acceptance of BL (Alhramelah and Alshahrani, 2020; Olabisi et al., 2017; Songsangyos et al., 2016). For instance, based on TAM model, prior studies have indicated that perceived ease of use, perceived usefulness and subjective norms are important factors positively affecting learners’ attitudes toward BL (Songsangyos et al., 2016; Tselios et al., 2011; Yu et al., 2023), but perceived usefulness of undergraduate students is more relevant to the acceptance of BL courses than perceived ease of use (Songsangyos et al., 2016). According to UTAUT model, scholars have found that performance expectance, effort expectance, social influence, facilitating conditions, hedonic motivation, price value and habit have varying degrees of impact on students’ behavioral intention to accept BL (Alhramelah and Alshahrani, 2020; Azizi et al., 2020; Olabisi et al., 2017). Additionally, the influence of other significant predictors also have been identified, such as the positive effect of pedagogy fitness, technology affinity and institutional readiness (Antwi-Boampong, 2020), and learning atmosphere (Zhao, 2022), as well as the negative impact of diverse online resources and excessive interference information (Zhao, 2022). As the research goes deeper, factors that influence learners’/students’ adaptability, satisfaction, SEF and learning effectiveness in BL have been widely explored. For example, Yang and Pu (2022) found that contextual factors, SEF, LM positively influence the adaptability of BL in non-English major college students. Chen and Yao (2016) indicated that perceived e-learner satisfaction of BL is influenced by the interplay of learners, instructors, courses, technology, design and environment. Wei et al. (2022) demonstrated that college students’ academic SEF of BL is primarily shaped by the personal, interpersonal and environmental factors. Lv and Li (2024) suggested that performance and effort expectations, hedonic motivation, and external facilitating conditions positively influence the BL effectiveness of college students through their behavioral intention.

Moreover, scholars also paid attention to the continuance intention to use BL (CIBL) in high education. Among these studies, learners’ personal characteristics such LM, SEF and satisfaction are considered as the most important factors affecting willingness to continue using BL (Chen et al., 2022; Jiang and Liang, 2023), where LM is the driving forces, while SEF and satisfaction are its prerequisite. For instance, Yang et al. (2023) found that performance expectancy, intrinsic motivation and satisfaction can significantly influence the beginners’ CIBL in the BL environment, and satisfaction has a mediating effect. Al-Busaidi and Al-Shihi (2012) indicated that satisfaction is the most significant determinant of instructors’ intention to use LMS in BL. Chen et al. (2022) suggested that both satisfaction and SEF are the key determinants of learners’ continuous intention to adopt BL. Jiang and Liang (2023) demonstrated that LM and SEF are able to significantly influence EFL students’ CIBL in a SPOC-based BL environment. Additionally, individual characteristics such as computer anxiety (Al-Busaidi and Al-Shihi, 2012), learning attitude (Birbal et al., 2018), perceived behavioral control and subjective norms (Baranova et al., 2022), technology experience and personal innovativeness (Al-Busaidi, 2013; Al-Busaidi and Al-Shihi, 2012), and performance expectancy, perceived usefulness and ease of use (Nikolopoulou and Zacharis, 2023) also exert significant impacts on learners’ CIBL. Moreover, the impact of external situational factors from instructors, the BL courses and learning platform on learners’ CIBL have been discussed. For example, Al-Busaidi and Al-Shihi (2012) put forward that characteristics from LMS and organizations, including information quality, management support, and incentives policy and training, can determine instructors’ behavioral use of LMS in BL via satisfaction. Chen et al. (2022) emphasized the importance of the course quality and technical support to learners’ acceptance of the BL system. Jiang and Liang (2023) examined the impact of the situational factors on EFL students’ continuance intention of BL by considering English teachers’ teaching, English curriculum satisfaction, SPOC learning platform assurance. Recently, scholars have paid attention to the factors influencing learners’ CBBL. For example, Zacharis and Nikolopoulou (2022) show that facilitating conditions and learning value can directly affect the leaners’ actual use of E-learning platform.

### 2.2 The SOR model

The SOR theory has developed as an extension of the S-R theory, which is first proposed in the research field of environmental psychology by Mehrabian (1974). The SOR model comprises three fundamental components: stimulus, organismic impact, and response, which explains that a range of external factors can serve as stimuli (S), subsequently influencing the internal state of an organism (O) and thereby affecting its individual response (R) (Mehrabian, 1974). Recent years, the SOR theory has been introduced and extensively applied within the domain of education, and it is used to explore how internal and external factors affect learners’ satisfaction, learning outcomes, LET and collaboration learning. For instance, in terms of satisfaction, Zhang et al. (2021) combined SCCT and SOR model to understand how social support systems and interaction relationships stimulate SEF and generic skills, and ultimately influence students’ satisfaction. Jung et al. (2021) investigated how centralized and peer-to-peer surveillance affect the satisfaction of group work through perceived surveillance and stress. He et al. (2022) found that learning attitude plays a mediating role in the impact of practical training courses’ features and SEF on students’ satisfaction. Regarding leaning outcomes and engagement, Fu et al. (2021) employed SOR framework confirming that smartphone overuse stimulates university students’ health problems of insomnia, nomophobia and poor eyesight, which can further negatively impact their academic performance. Pan et al. (2024) revealed that interactions in online learning environments significantly enhance learners’ perceptions of the usefulness and ease of use of such platforms, thereby ultimately positively influencing their learning effectiveness. Yang J. et al. (2021) investigated how perceived closeness, peer referents and perceived control improve students’ enthusiasm for e-learning through SEF and wellbeing based on SOR theoretical model. With respect to collaboration learning, Zhai et al. (2023) show that privacy concerns can lead to knowledge hiding perception among students, thereby negatively impacting their online collaboration, while perceived supervisory support moderates this effect. Moreover, some scholars have also focused on the factors influencing students’ continuance intention under the SOR framework. For instance, Yang et al. (2019) analyzed the how perceived learning support, self-management and peer influence affect college students’ M-learning continuance via LET. Zhao et al. (2020) found that the interactive features of the technological environment and media richness enhance telepresence, while sociability and media richness foster social presence, both of which can increase the intention to continue using MOOCs. Chang (2022) show that flow experiences and satisfaction mediate the relationship between students’ perceived ease of use and usefulness in ATM model and M-learning continuance. Zhou and Fang (2024a) examined how the characteristics of short video recommendations stimulate students’ perceived usefulness, LET and LM, which in turn affect their continuance intention of utilizing short videos.

Overall, extant scholars have conducted extensive research on BL and its determinants, meanwhile, the significant achievements have been made by extending the SOR model into the educational context. Nevertheless, some limitations remain evident. First, although the existing studies have widely explored the driven forces of initial adoption of BL based on ATM or UTAUT model (Alhramelah and Alshahrani, 2020; Olabisi et al., 2017; Songsangyos et al., 2016), the antecedents of adaptability, satisfaction, and SEF and learning effectiveness in the BL environment (Chen and Yao, 2016; Lv and Li, 2024; Wei et al., 2022; Yang and Pu, 2022), the factors influencing college students’ continuance participation in a BL course have been largely overlooked. In addition, although several prior literature has investigated the factors of CIBL from the individual and external contextual perspective (Al-Busaidi, 2013; Al-Busaidi and Al-Shihi, 2012; Chen et al., 2022; Jiang and Liang, 2023), the majority of these studies have focused on the impact of individual characteristics and external contextual factors on CIBL separately. Most importantly, they rarely consider the impact of BCC, and further integrate BCC, ITS and internal individual’s features into a whole framework to conduct a comprehensive analysis. Second, previous studies have employed the SOR framework to investigate the stimulations of college students’ satisfaction (He et al., 2022; Zhang et al., 2021), learning outcomes (Fu et al., 2021; Pan et al., 2024), learning effect and engagement (Yang J. et al., 2021), collaboration learning (Zhai et al., 2023) and continuance intention (Chang, 2022; Yang et al., 2019; Zhou and Fang, 2024a), few scholars empirically reveal what factors will stimulate continuance behavior among college students in the MOOC-based BL environment from the SOR model perspective, and what role do individual characteristics, such as LM, LET and SEF, will play in this influencing mechanism. Therefore, based on the SOR model, this study constructs an integrated research model of *“Contextual facilitators (S)–Individual characteristics (O)–Continuance behavior (R)”* to explore the determinants of college students’ CBBL by integrating BCC, ITS and individual characteristics into a holistic analysis framework. Specifically, this paper conducts an in-depth analysis of the influencing mechanism by considering BCC and ITS as stimuli, individual characteristics—including LM, SEF, and LET—as organisms, and college students’ CBBL as the final response.

## 3 Hypotheses and research model

### 3.1 BCC and individual factors

Extant scholars have emphasized the importance of the learning environment for learners, distinguishing it into “physical and social environments within a classroom setting” (Wu et al., 2010). Regarding to contextual factors in the BL environments, scholars define them as non-learner characteristics that make up the learning environment and support students’ learning activities (Yang S. et al., 2021). Compared with other courses, the BL courses exhibit notable features such as flexibility, rich resources, personalized learning, and diverse interactions (Yen and Lee, 2011; Yu et al., 2023). Therefore, the BL courses can cultivate several key non-learner characteristics, which can directly influence college students’ learning experiences and overall state. On one hand, the BL courses use smart devices, online platforms, and LMS to create flexible learning spaces, breaking the temporal and spatial constraints of traditional learning. This flexibility allows students to manage their own learning pace, enhancing their sense of control and positively impacting their motivation and confidence. On the other hand, the BL courses emphasize the interactions among students, as well as between students and teachers. Generally, students are encouraged to actively engage in the BL process through discussions, positive feedback and collaboration. They can receive support timely when facing challenges, thereby reducing frustration, maintaining motivation, and enhancing self-efficacy. Previous research has also indicated that the English BL courses can positively impact learners’ motivation and self-efficacy (Yang and Pu, 2022). Purarjomandlangrudi and Chen (2020) found that students’ perceptions of course characteristics like a sense of presence, identity, and purpose have a positive impact on their online interactions and learning engagement. He et al. (2022) indicated that characteristics of practical training course can significantly influence college students’ satisfaction through learning attitudes. According to these arguments, the following hypotheses are proposed:

*H1a*: BCC is positively associated to college students’ LM in the BL environment.

*H1b*: BCC is positively associated to college students’ SEF in the BL environment.

*H1c*: BCC is positively associated to college students’ LET in the BL environment.

### 3.2 ITS and individual factors

Social constructivism views learning as a participatory social process, where interpersonal interactions facilitate the exchange of knowledge (Moll and Greenberg, 1992). Instructors are one of the key implementers in the BL environment (Feng et al., 2023), the interaction between instructors and students is a kind of important interpersonal relationship. Thus, ITS can be regarded as another critical contextual factor of affecting students’ learning state. ITS refers to students’ perception that their instructors show genuine care for their learning and are willing to help them solve problem during the learning process (Trickett and Moos, 1973). ITS includes the beneficial social teaching practices, such as providing learning resources, guiding the use of leaning platforms and tools, clarifying course material, correcting misunderstandings, offering timely feedback, and giving constructive advice on performance (Wei et al., 2022; Zheng et al., 2024). In the BL setting, the accessibility and variety of online resources give instructors opportunities to present content in diverse ways. Instructors can focus more on their core strengths—designing engaging and well-structured courses (Lungu, 2013). They can interact with students through multiple channels, such as using smart devices to present content, organizing more classroom activities, and providing more timely and specific feedback, which in turn, positively influence students’ LM, SEF, and LET. According to the social cognitive theory, external factors (i.e., teacher) can significantly influence learners’ SEF and motivation (Bandura, 1986). Prior studies also have shown that students who perceive greater support from their instructors tend to feel more confident in their abilities and engage more actively in subjects of math and science (Rice et al., 2013). ITS can contribute to foster and sustain both SEF and engagement in student-centered learning (Lee and Baird, 2021). Moreover, whether in the BL environment or face-to-face instruction, the perceived support from instructors is an important antecedent of enhancing students’ self-confidence (Gutiérrez and Tomás, 2019; Wei et al., 2022). Thus, we propose the hypotheses as follows:

*H2a*: ITS is positively associated to college students’ LM in the BL environment.

*H2b*: ITS is positively associated to college students’ SEF in the BL environment.

*H2c*: ITS is positively associated to college students’ LET in the BL environment.

### 3.3 Individual factors and college students’ CBBL

#### 3.3.1 The impact of LM on college students’ CBBL

Motivation refers to the psychological state in which an individual engages in certain activities spontaneously and sustain behavior, without being constrained by coercion (Ryan and Deci, 2000). Self-determination theory suggests that motivation comes in both intrinsic and extrinsic forms. Specifically, intrinsic motivation is self-determined, referring to an individual’s engagement in a particular behavior out of internal interest and pure enjoyment (Valerio, 2012), while extrinsic motivation is defined as individual behavior influenced by its perceived values and the anticipated benefits of the action (Buzdar et al., 2017). Prior studies indicated that both intrinsic and extrinsic motivations are the significant antecedents of behavioral intention of educational system or technology (Meng and Li, 2023). In the BL process, intrinsic motivation refers to college students’ interest in course content, characterized by a strong desire to explore and the expectation of experiencing satisfaction and a sense of achievement during the learning process, while extrinsic motivation is that students are influenced by external factors when they are engaged in the BL courses, such as being driven by specific course assignments, academic rewards, scholarships, prize, opportunities of postgraduate recommendation and peer competition. Extant studies have proposed that LM is a crucial factor influencing achievement-related behavior (Yang and Pu, 2022). In the BL environment, LM is closely linked to students’ learning autonomy, ability to complete online and offline tasks independently, choice of learning strategies, as well as the interaction with instructors. Thus, if the LM is higher, the likelihood of students to continue participating in the BL courses is higher. Moreover, prior studies have also provided many evidence to support the positive relationship between LM and learners’ continuance behavior (Jiang and Liang, 2023). Thus, the hypothesis is proposed as follows:

*H3a:* LM is positively associated to college students’ CBBL.

#### 3.3.2 The impact of SEF on college students’ CBBL

SEF refers to people’s belief in their ability to control events that affect their lives, which can be traced back when any psychological or behavioral have changed (Bandura, 1986). In the BL environment, SEF refers to students’ confidence in their ability to successfully complete learning tasks, master course content, and navigate the combined online and offline learning model. Previous studies have emphasized the critical role of SEF in technology-based learning (Hatlevik and Bjarnø, 2021). For instance, Hatlevik and Bjarnø (2021) indicated that students’ SEF in information technology positively influenced their willingness to invest time in tasks within a technology-based learning environment. Yang and Pu (2022) suggested that an individual’s belief in their ability to manage and navigate changes is crucial for adaptation, and students with a high level of SEF are more likely to adjust effectively to the BL model. Lv and Li (2024) found that SEF can contribute to college students achieve better effectiveness of BL. According to these studies, we further explore the connection between college students’ SEF and their continuance behavior in the BL environment. High SEF encourages students to proactively make learning plans and choose suitable strategies for BL courses. When encountering technical issues or academic challenges, students with high SEF are more likely to adopt positive measures for independent problem-solving, which will reduce the likelihood of giving up and enhancing their persistence. Additionally, students with strong SEF are more receptive to feedback from teachers and peers, viewing it as a valuable opportunity for improvement. This positive feedback loop enables them to continuously optimize learning strategies. Previous studies have confirmed the positive impact of SEF on learners’ continuance behavior. For example, Alamri (2022) found that academic self-efficacy is a key factor influencing students’ learning persistence on MOOCs adoption during the COVID-19 pandemic. Therefore, we put forward the following hypothesis:

*H3b:* SEF is positively associated to college students’ CBBL.

#### 3.3.3 The impact of LET on college students’ CBBL

LET refers to the extent of students’ behavioral participation and emotional experience during the initiation and completion of learning activities (Halverson and Graham, 2019). In context of BL, LET refers to the time, effort, and emotional investment that students put into the learning process. Previous studies have found the positive impact of LET on learners’ persistence. For instance, Yang et al. (2013) discovered that the participation in MOOC learning activities like posting in forums can significantly decrease the likelihood of leaners’ dropouts. Breslow et al. (2013) show that the majority of students who earned a certificate in the course had actively participated in posting on the course forums, indicating that this level of LET is a more reliable predictor of completing MOOCs. Pursel et al. (2016) found that taking part in activities like viewing lectures, can demonstrate significant predictive validity for the successful completion of MOOCs. Jung and Lee (2018) indicated that LET can positively affect the intention to complete a MOOC. Therefore, it can be believed that LET has a positive impact on students’ continuance behaviors in the BL situation. In the process of BL, actively engaged students tend to develop more positive learning experiences—including academic satisfaction and achievement—which foster sustained content engagement while reinforcing their BL identity and persistence intentions. Moreover, students with high LET are more likely to actively participate in group discussions and collaborative learning, thereby enhancing their interactions with peers and instructors. This participation not only helps students better understand and master knowledge, improving learning outcomes, but also motivates them to continue engaging in BL. Consequently, the following hypothesis is proposed:

*H3c*: LET is positively associated to college students’ CBBL.

### 3.4 The mediating role of individual factors

According to the SOR framework, the impact of stimuli on user’s behavior can be mediated by the individual’s internal state (Yang J. et al., 2021). Based on the arguments above, this study suggests that the associations between external contextual facilitators and college students’ continuance behavior in the BL settings might be mediated by their LM, SEF, and LET. In the domain of educational research, some scholars have confirmed the mediating role of LM, SEF, and LET. For instance, Chu and Tsai (2009) indicated that adult learners’ SEF in using the internet exerted an indirect impact on the association between internet usage and their preferences toward CILE. Shea and Bidjerano (2010) demonstrated that SEF played a mediating role in the relationship between external environmental factors-digital infrastructure and social support-on students’ self-regulatory behaviors in online learning. Bukhari et al. (2014) demonstrated that LM is a crucial mediating factor in online learning context, and its mediating effect in the relationship between technology features and learning behaviors become more significant after they were trained. Jung and Lee (2018) suggested that factors such and teaching presence and perceived usefulness could influence learners’ persistence in online learning situations through LET. Yang and Pu (2022) shown that the influence of contextual factors on the adaptability of learners who are majored in non-English is significantly mediated by SEF and LM. Therefore, the following hypotheses are put forward:

*H4*: The relationship between BCC and college students’ CBBL is mediated by LM (H4a), SEF (H4b) and LET (H4c).

*H5*: The relationship between ITS and college students’ CBBL is mediated by (H5a), SEF (H5b) and LET (H5c).

**Figure 1:**
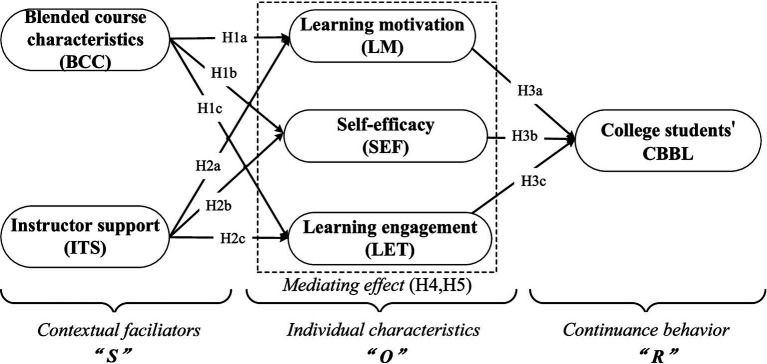
The research model.

Figure 1 presents the integrated SOR model that investigates the key factors influencing college students’ CBBL, which includes five latent variables, one observed variable and a total of 11 hypotheses.

## 4 Methodology

### 4.1 Constructs and measurements

According to the hypotheses and the integrated SOR model proposed above, the questionnaire concerning the determinants of college students’ CBBL was designed, which included the participants’ demographic information, such as gender, grade and major, and the measurement of the six constructs with the Likert-type five-point scale, namely blended course characteristics (BCC), instructor support (ITS), learning motivation (LM), self-efficacy (SEF), learning engagement (LET), and college students’ continuance behaviors of blended learning (CBBL).

#### 4.1.1 BCC variable

To measure BCC variable, we used eight items, and modified based on existing literature, such as He et al. (2022) and Moely and Ilustre (2014). Each item and its definition are presented in Table 1.

**Table 1:** Measurement of BCC variable.

Variable	Items and definitions	
Blended course characteristics (BCC)	BCC1	The BL courses on the Chinese university MOOCs platform allow me to flexibly manage the time and pace of online learning.
	BCC2	The BL courses on the Chinese university MOOCs platform offer me a more diverse range of learning options and pathways.
	BCC3	The BL courses on the Chinese university MOOCs platform expose me to a variety of learning resources, including videos, audio, images, and animations.
	BCC4	The BL courses on the Chinese university MOOCs platform allow me to quickly access study materials such as textbooks, lecture slides, study guides, and reference materials.
	BCC5	The BL courses on the Chinese university MOOCs platform can provide personalized support based on my interests and learning progress.
	BCC6	The BL courses on the Chinese university MOOCs platform allow me to choose a suitable time for review and reinforcement.
	BCC7	The BL courses allow me to engage in teacher-student and student–student interactions through the Chinese university MOOCs platform.
	BCC8	The BL courses provide me with more opportunities to interact with teachers and classmates through the Chinese university MOOCs platform.

#### 4.1.2 IST variable

According to extant studies, seven items were utilized to measure support from instructors, and the items were the scales from Wei et al. (2022) and Walker and Fraser (2005). Table 2 illustrates the items in detail.

**Table 2:** Measurement of IST variable.

Variable	Items and definitions	
Instructor support (ITS)	ITS1	Instructors can address the questions I encounter during the BL process on the Chinese university MOOCs platform at the appropriate time.
	ITS2	Instructors can help me identify and analyze the issues that arise during the BL process on the Chinese university MOOCs platform.
	ITS3	Instructors can provide important feedback on the assignments I submit during the BL process on the Chinese university MOOCs platform.
	ITS4	Instructors can provide detailed answers to the questions that I propose during the BL process on the Chinese university MOOCs platform.
	ITS5	Instructors encourage me to engage in the BL courses on the Chinese university MOOCs platform.
	ITS6	If I encounter problems in the BL courses on the Chinese university MOOCs platform, it’s easy to contact my teachers and get help.
	ITS7	Instructors provided positive or negative feedback on my performance in the BL courses on the Chinese university MOOCs platform.

#### 4.1.3 LM, SEF, and LET variables

The variables of LM, SEF, and LET are widely investigated in existing studies. This study used four, three and 11 items to evaluate LM, SEF, and LET, separately. Specifically, the scales of LM were mainly adopted from Buzdar et al. (2017) and Yang and Pu (2022). To measure SEF, the scales were utilized from Chen et al. (2022) and Jiang and Liang (2023). And the scales of measuring LET primarily were employed from Pursel et al. (2016) and Jung and Lee (2018). Table 3 shows the scales and items.

**Table 3:** Measurement of LM, SEF, and LET variables.

Variables	Items and definitions	
Learning motivation (LM)	LM1	I hope that engaging in the BL courses on the Chinese university MOOCs platform will provide me with more learning opportunities.
	LM2	I hope that engaging in the BL courses through the Chinese university MOOCs platform will enhance the flexibility and enjoyment of my learning experience.
	LM3	I can complete learning tasks and participate in discussions and interactions without supervision.
	LM4	Engaging in the BL courses on the Chinese university MOOCs platform allows me to better manage my study time and achieve better learning outcomes.
Self-efficacy (SEF)	SEF1	I can effectively adapt to the BL courses on the Chinese university MOOCs platform and complete the related assignments on time.
	SEF2	I can effectively achieve the learning objectives of the courses through BL model on the Chinese university MOOCs platform.
	SEF3	I am confident in overcoming the various challenges and difficulties faced in implementing BL model on the Chinese university MOOCs platform.
Learning engagement (LET)	LET1	I can adapt to and comply with the rules of the BL courses through the Chinese university MOOCs platform.
	LET2	I am able to maintain focus during both online and offline learning processes while using the Chinese university MOOCs platform for BL.
	LET3	I can complete the assignments assigned in the BL courses on time.
	LET4	I feel excited during the process of engaging in the BL courses on the Chinese university MOOCs platform.
	LET5	I am very interested in BL model through the Chinese university MOOCs platform.
	LET6	I try to find information related to the BL courses through other channels, such as journal articles and magazines.
	LET7	When I engage in the BL courses, I ask myself questions to ensure that I understand the course content.
	LET8	I read additional materials to gain deeper insights into the BL courses on the Chinese university MOOCs platform

#### 4.1.4 College students’ CBBL variable

Existing scholars have conducted an extensively studies on learners’ continuance behavior, and the majority of studies adopt the scales from Venkatesh et al. (2003) and Venkatesh et al. (2012) to measure continuance behavior. According to these prior studies, this study also uses this scale containing four items to measure college students’ CBBL. Table 4 presents the items and definitions.

**Table 4:** Measurement of college students’ CBBL variable.

Variable	Items and definitions	
College students’ continuance blended learning behavior (CBBL)	CBBL1	I have frequently learned the BL courses through the Chinese university MOOCs platform last month.
	CBBL2	I have been using the Chinese university MOOCs platform for BL almost every week over the past month.
	CBBL3	I have learned the BL courses through the Chinese university MOOCs platform frequently during the past month.
	CBBL4	I have spent much time on the BL courses on the Chinese university MOOCs platform in the past month.

### 4.2 Data collection

We carried out an online survey via Sojump.com. A random sampling technique was employed to select college students who had taken blended courses on Chinese university MOOCs platform as the research subjects, and distributed questionnaires to them. During the survey process, all participants were informed that the collected data would be used solely for academic research, and their personal information would be fully protected. Moreover, the sample data was processed as follows: First, we set the question that “Have you ever studied blended courses through Chinese MOOCs platform?” and deleted records that answered “no”; Second, we deleted records that was taken <30 s to complete the questionnaire. After that, 466 valid samples was collected.

### 4.3 Data analysis

SEM is a statistical method used to analyze relationships between latent variables and observed variables (Barrett, 2007). It integrates factor analysis and path analysis, allowing for the simultaneous handling of measurement errors and the estimation of causal relationships between variables. SEM consists of two main components: the measurement model and the structural model. The measurement model, also known as confirmatory factor analysis (CFA), describes the relationships between latent variables and their observed variables. The structural model describes the causal or path relationships between latent variables. In the process of quantitative analysis, SPSS 29 is used to conduct descriptive statistics, the reliability and validity of the questionnaire and variables, and exploratory factor analysis (EFA). AMOS 26 is utilized to conduct the confirmatory factor analysis (CFA) and construct a structural model. The evaluation indexes of model fit usually include chi-square freedom ratio (*χ*^2^/df), root mean square error of approximation (RMSEA), standardized root mean square residual (SRMR), comparative fit index, (CFI), goodness of fit index (GFI) and Tucker-Lewis index (TLI) (Bagozzi and Yi, 1988; Yu et al., 2023). Moreover, AMOS 26 is also used to test the mediating effects of LM, SEF, and LET by performing percentile bootstrapping and bias-corrected percentile bootstrapping (Taylor et al., 2008).

## 5 Results

### 5.1 Descriptive analysis

Table 5 illustrates the demographic information of participating students. These demographic characteristics include gender, grade and major. Among the 466 participants, the number of male was more than female, the percentage of which were 59.87 and 40.13%, respectively. Regarding to the grade, students of the four grades all participated, but the majority were sophomore and junior students, accounting for 72.32% of the total. In terms of major distribution, the number of students in sciences and liberal arts were relatively balanced, with sciences accounting for 56.22% and liberal arts accounting for 43.78%.

**Table 5:** Demographic information of respondents.

Characteristics	Items	Number	Percentage
Gender	Male	279	59.87%
	Female	187	40.13%
	Total	466	100.00%
Grade	Freshman	58	12.45%
	Sophomore	174	37.34%
	Junior	163	34.98%
	Senior	71	15.24%
	Total	466	100.00%
Major	Science	262	56.22%
	Liberal arts	204	43.78%
	Total	466	100.00%

### 5.2 Reliability and validity tests

From the results shown in Table 6, it can be found that the Kaiser-Meyer-Olkin (KMO) value was 0.892, above the 0.7 threshold. Moreover, the *χ*^2^ value of Bartlett’s test of Sphericity is 8585.150 and *p* < 5% significance level, suggesting that factor analysis is suitable for subsequent use. Therefore, in what follows, we conduct EFA and CFA to test the reliability and validity of the questionnaire.

**Table 6:** KMO and Bartlett test.

KMO		0.892
Bartlett’s test of sphericity	*χ*^2^ value	8585.150
	Degrees of freedom	561
	*p*-value	0.000

Before the analysis of EFA and CFA, this study guarantees content validity through rigorous refinement and modification based on literature reviews and pre-project surveys to ensure the construct validity of the scale. Table 7 presents the results of EFA and CFA. Regarding EFA, the cumulative variance of the first 6 factors is 62.85%, indicating that these factors can explain a significant amount of information from the original variables. As indicated in Table 8, each item’s factor loading exceeds 0.5, showing that there is a good correspondence between factors and terms. Thus, the scale of college students’ CBBL and its determinants developed in our study has good convergent validity (Zaichkowsky, 1985). With respect to CFA, Cronbach’s *α*, CR and AVE are used to assess the reliability of the scale. As shown in Table 7, it can be found that the Cronbach’s α values for BCC, ITS, LM, SEF, LET and college students’ CBBL are consistently more than 0.7, showing that the internal consistency of the questionnaire is very good and the data reliability is high (Fornell and Larcker, 1981). Moreover, each latent variable’ CR value exceeded 0.8, and their AVE values surpassed 0.5. Demonstrating that the questionnaire designed in this study has good convergent validity and construct validity (Fornell and Larcker, 1981).

**Table 7:** Results of reliability and validity tests of questionnaire.

Variables	Items	Factor loadings	SMC	CR	AVE	Cronbach’s α
BCC	BCC1	0.881	0.776	0.959	0.743	0.953
	BCC2	0.867	0.752			
	BCC3	0.875	0.766			
	BCC4	0.847	0.717			
	BCC5	0.858	0.736			
	BCC6	0.864	0.746			
	BCC7	0.851	0.724			
	BCC8	0.854	0.729			
ITS	ITS1	0.713	0.508	0.875	0.500	0.839
	ITS2	0.721	0.520			
	ITS3	0.694	0.482			
	ITS4	0.692	0.479			
	ITS5	0.740	0.548			
	ITS6	0.707	0.500			
	ITS7	0.682	0.535			
LM	LM1	0.842	0.709	0.909	0.713	0.877
	LM2	0.834	0.696			
	LM3	0.854	0.729			
	LM4	0.848	0.719			
SEF	SEF1	0.889	0.790	0.923	0.800	0.883
	SEF2	0.894	0.799			
	SEF3	0.900	0.810			
LET	LET1	0.705	0.497	0.893	0.512	0.868
	LET2	0.729	0.531			
	LET3	0.701	0.491			
	LET4	0.712	0.507			
	LET5	0.729	0.531			
	LET6	0.686	0.471			
	LET7	0.737	0.543			
	LET8	0.722	0.521			
CBBL	CBBL1	0.693	0.480	0.804	0.506	0.705
	CBBL2	0.738	0.545			
	CBBL3	0.707	0.500			
	CBBL4	0.706	0.498			

**Table 8:** Discriminant validity test results.

Variables	ITS	BCC	SEF	LET	LM	CBBL
ITS	**0.707**					
BCC	0.009	**0.862**				
SEF	0.161	0.178	**0.894**			
LET	0.115	0.057	0.028	**0.715**		
LM	0.118	0.195	0.053	0.024	**0.845**	
CBBL	0.283	0.190	0.192	0.150	0.190	**0.711**

The diagonal numbers in bold are the values of the square root of the average variance extraction (AVE).

Additionally, the study further examined the discriminant validity of the questionnaire. From the results of Table 8, it can be found that all the square root of AVE displayed on the dialog line, namely, 0.707, 0.862, 0.894, 0.715, 0.845, and 0.711, were greater than the correlation values between variables presented in the non-dialog, indicating that the discriminant validity of the questionnaire meet the requirements (Fornell and Larcker, 1981).

### 5.3 Hypotheses testing

Table 9 presents the results of the overall model fit. The values of *χ*^2^/df and RMSEA were 1.075 and 0.012, both of which were less than the good fit level (*χ*^2^/df < 3; RMSEA<0.08). Moreover, all the values of GFI, AGFI, CFI and TLI were more than 0.9, and the value of SRMR was 0.032, which was the acceptable fit level (SRMR < 0.5). These results indicate that the fit indices of the constructed model meets the established standards and the questionnaire data demonstrates an overall good fit (Hu and Bentler, 1998).

**Table 9:** Fitness test of the model.

Indicators		*χ*^2^/df	RMSEA	GFI	AGFI	CFI	SRMR	TLI
Evaluation criterion	Good	<3.0	<0.08	>0.9	>0.9	>0.9	Close to 0	>0.9
	Acceptable	3.0–5.0	0.08–0.1	0.7–0.9	0.7–0.9	0.7–0.9	<0.5	0.7–0.9
Estimated value		1.075	0.012	0.940	0.931	0.995	0.032	0.994

**Figure 2:**
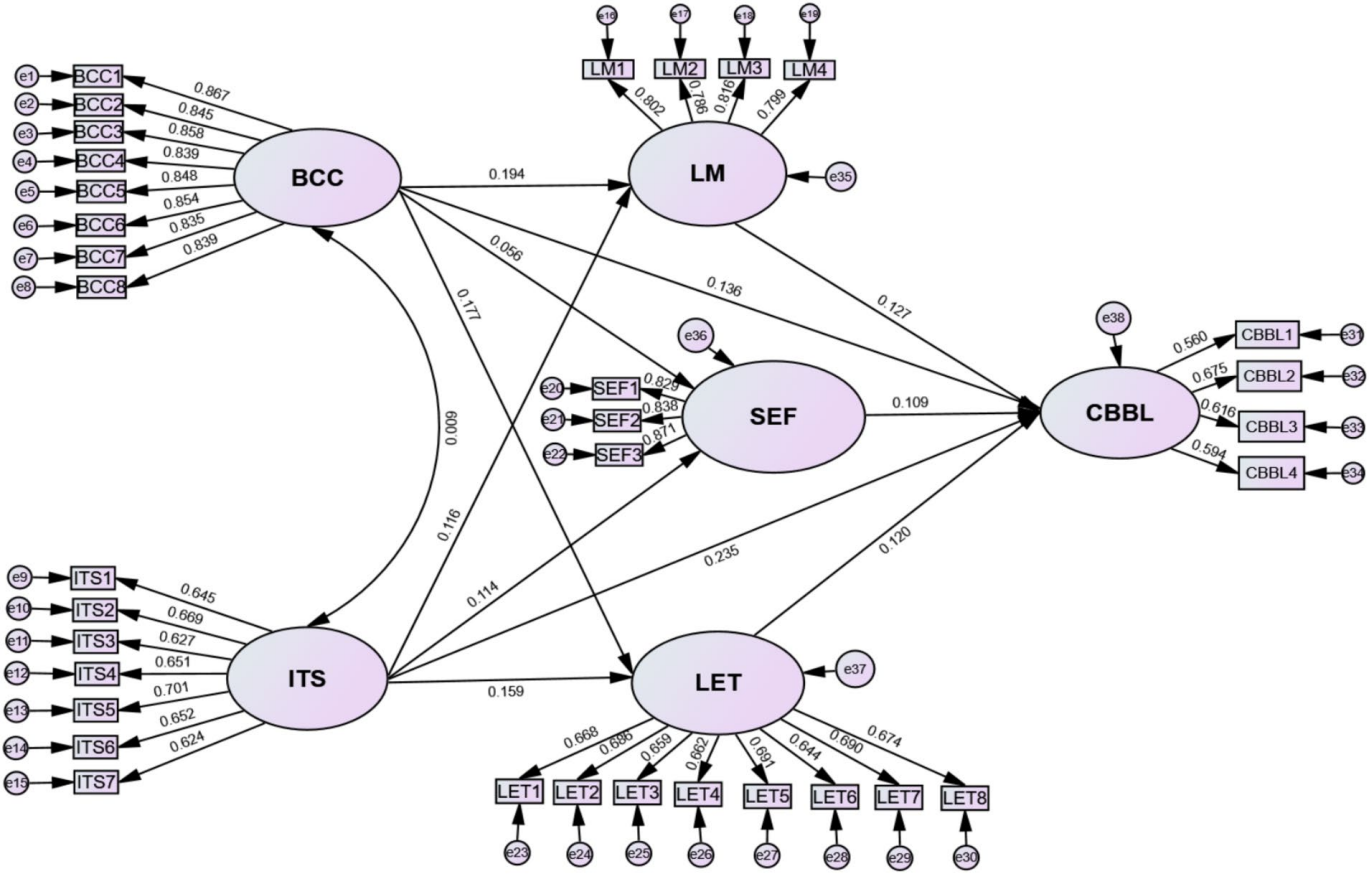
SEM with the standardized coefficients and influencing paths.

The results of SEM are presented in Table 10, and Figure 2 is the SEM with standardized coefficients of variables. First of all, we examined the direct effects of BCC and ITS on college students’ CBBL. Both BCC and ITS positively affect college students’ CBBL (*ꞵ* = 0.188, *p* = 0.014; *ꞵ* = 0.282, *p* < 0.01), and the impact of ITS was greater than BCC. After that, each path has been examined. As indicated in Table 10, it can be found that, excepting for the path of SEF ← BCC was not significant (*ꞵ* = 0.056, *p* = 0.247), other paths were significant at 1% or 5% levels. Specifically, BCC can significantly enhance college students’ LM (*ꞵ* = 0.194, *p* < 0.01) and LET (*ꞵ* = 0.177, *p* < 0.01), and ITS can positively affect college students’ LM (*ꞵ* = 0.116, *p* = 0.025), SEF (*ꞵ* = 0.114, *p* = 0.030) and LET (*ꞵ* = 0.159, *p* = 0.003), while college students’ CBBL can be significantly influenced by their LM (*ꞵ* = 0.127, *p* = 0.026), SEF (*ꞵ* = 0.109, *p* = 0.049) and LET (*ꞵ* = 0.120, *p* = 0.037). These results indicate that all proposed hypotheses are supported excepting for H2b. Moreover, among these relationships, BCC exerts a greater influence on college students’ LM, while ITS has a strongest impact college students’ LET, and their CBBL are primarily influenced by LM and LET.

**Table 10:** Results of hypotheses testing.

Hypotheses	Path	Regression weights	Std. regression weights	S.E.	C.R.	*p*-value	Results
H1a	LM ← BCC	0.161	0.194	0.041	3.957	***	Support
H1b	SEF ← BCC	0.060	0.056	0.052	1.157	0.247	Reject
H1c	LET←BCC	0.138	0.177	0.039	3.569	***	Support
H2a	LM ← ITS	0.135	0.116	0.060	2.234	0.025	Support
H2b	SEF ← ITS	0.168	0.114	0.077	2.174	0.030	Support
H2c	LET ← ITS	0.173	0.159	0.058	2.995	0.003	Support
H3a	CBBL ← LM	0.096	0.127	0.043	2.231	0.026	Support
H3b	CBBL ← SEF	0.064	0.109	0.033	1.967	0.049	Support
H3c	CBBL ← LET	0.096	0.120	0.046	2.083	0.037	Support

***indicates a significance level of 1%.

Furthermore, bootstrap analysis was also used to test the mediating effects of individual’ attributes between BCC and CBBL as well as ITS and CBBL. As shown in Table 11, the effects of BCC on college students’ CBBL through LM and LET were 0.025 and 0.021, respectively, and the 95% confidence intervals (CI) were (0.003–0.038) and (0.001–0.040), and 0 were excluded, suggesting that LM and LET mediate the relationships between BCC and college students’ CBBL, but the mediating effect of SEF was insignificant. Thus, H4a and H4c are supported, but H4b is rejected. Moreover, the paths from ITS to college students’ CBBL through LM, SEF, and LET were 0.015, 0.012 and 0.019, respectively, the CI of which were (0.001–0.043), (0.001–0.034), and (0.001–0.052), and 0 were not contained, indicating that the mediating roles of LM, SEF, and LET in the associations between ITS and LM, SEF, and LET were significant. Therefore, H5a, H5b and H5c are supported.

**Table 11:** Bootstrap results of mediating effects for LM, SEF, and LET.

Hypotheses	Model path	Estimated coefficients	*p*-value	95%CI		Results
				BootLLCI	BootULCI	
H4a	BCC → LM → CBBL	0.025	0.008	0.003	0.038	Support
H4b	BCC → SEF → CBBL	0.006	0.173	−0.002	0.017	Reject
H4c	BCC → LET→CBBL	0.021	0.030	0.001	0.040	Support
H5a	ITS → LM → CBBL	0.015	0.036	0.001	0.043	Support
H5b	ITS → SEF → CBBL	0.012	0.029	0.001	0.034	Support
H5c	ITS → LET → CBBL	0.019	0.032	0.001	0.052	Support

## 6 Discussion and conclusion

### 6.1 Discussion

Adopting the SOR framework and social cognitive theory as the foundation, this paper developed a theoretical model of *“Contextual facilitators–Individual characteristics–Continuance behavior,”* to explore the multiple influencing factors affecting college students’ CBBL, wherein external contextual facilitators–BCC and ITS–were stimulus, individual attributes–LM, SEF, and LET–were organism, and college students’ CBBL was response. SPSS 29 software and AMOS 26 software were utilized to effectively conduct quantitative analysis and test hypotheses proposed within the framework of SEM. The empirical results indicate that the following eight of the nine hypotheses proposed in the study framework were validated: BCC → LM, BCC → LET, ITS→LM, ITS→SEF, ITS→LET, LM → CBBL, SEF → CBBL, LET→CBBL. LM (*β* = 0.127, *p* = 0.026), SEF (*β* = 0.109, *p* = 0.049), LET (*β* = 0.120, *p* = 0.037) have a positive impact on college students’ CBBL. These results indicate that the integrated model in this study was valid, and 62.85% of the variance in college students’ CBBL variable could be explained by the three independent variables, i.e., LM, SEF, and LET. In another word, LM, SEF, and LET emerge as the key determinants influencing college students’ CBBL. This study not only extends this line of existing literature on college students’ continuance behavior in the BL environment by simultaneously considering the impact of external facilitators and individual factors, but also enhancing the explanatory power and the application range of the SOR model by successfully testing the hypotheses. The detailed findings and discussions are presented as follows:

1. BCC positively influences college students’ LM and LET, but has no significant impact on SEF.

The empirical results demonstrate that BCC has a positive impact on college students’ LM and LET in the BL environment. This significant influence of BCC on college students’ LM and LET may stem from its direct influence on learning task value and situational engagement. On one hand, the BL course design has a structured feature and can support college students to flexibly learn modular content in both online and offline environments (Yang and Pu, 2022; Yu et al., 2023). Moreover, the modular content and progressive learning challenges in BL courses can effectively stimulate students’ goal-oriented behaviors and enhance their LM by increasing course interesting and task value perception. On the other hand, the course’s multi-modal resource library, personalized learning support, and interactive task design, through the mechanism of optimizing cognitive resource allocation, significantly reduce the cognitive load pressure on college students. Meanwhile, relying on contextualized learning methods, it further deepens immersive on-site experience, thereby effectively increasing college students’ LET (Purarjomandlangrudi and Chen, 2020). However, our results indicate that the impact of BCC on SEF is insignificant, which is opposite to prior studies (Yang and Pu, 2022). The possible explanations may be that SEF usually stems from personal achievement experiences, social recognition and self-reflection, and more relies on the construction of one’s personal ability assessment system. Meanwhile, BCC may focus more on content attractiveness rather than ability cultivation. Therefore, even if BL courses have features such as flexibility, interaction and diversity, college students may still lack confidence in themselves due to insufficient perception of their personal abilities. Moreover, the cultivation of SEF has a cumulative effect, thus the short-term course characteristics as external stimuli are difficult to directly intervene in the formation of college students’ internal abilities.

2. ITS has a positive impact on college students’ LM, SEF, and LET, with the greatest impact on LET.

Our results reveal that ITS as an external stimulus can significantly influence college students’ psychological and cognitive states, and improve their LM, SEF, and LET. This result emphases the important role of teachers in the BL environment. On one hand, instructors’ support, such as timely feedback, personalized guidance, and encouragement of interaction, can enhance college students’ interest in BL learning and their intrinsic motivation (Yang and Pu, 2022). According to self-determination theory, when students feel supported by their teachers, their sense of autonomy, competence, and relatedness is fulfilled, thereby promoting an increase in their learning motivation (Sørebø et al., 2009). On the other hand, Teachers can help students gain experiences of learning success by providing mastery experiences, vicarious experiences and social persuasion, which helps students build the belief that they have the ability to complete tasks of BL courses, and thereby enhancing their self-efficacy in the BL process (Gutiérrez and Tomás, 2019; Wei et al., 2022). Finally, Teachers can enhance students’ classroom interaction and sense of belonging by providing abundant learning resources, promoting interaction, as well as showing encouragement, making them more willing to engage in the BL courses and further improving their in-depth understanding of the course content. Therefore, IST has a positive impact on college students’ behavioral engagement, emotional engagement, and cognitive engagement in all three aspects. Additionally, our results also indicated that ITS has a greater impact on LET than LM and SEF. It can be explained that the formation of LM requires going through a cognitive processing cycle of internalizing goals and reconstructing values, while the establishment of SEF depends on an iterative process of accumulating experience. Therefore, it needs a certain time for IST enhancing college students’ LM and SEF in the BL environment. However, it can produce immediate intervention effects on their LET.

3. LM, SEF, and LET are the critical determinants that significantly and positively influence college students’ CBBL.

Among the personal attributes, our results reveal that LM is the most significant predicator for college students’ CBBL. This result is consistent with previous studies on the relationship between LM and continuance learning (Jiang and Liang, 2023; Yang and Pu, 2022), which hold the views that motivation is a kind of pivotal catalyst for increasing learning enthusiasm and promoting studying independently. Thus, when students demonstrate a willingness to engage actively in BL activities on the Chinese MOOCs platform, they are likely to develop stronger persistence regarding their learning behavior. Moreover, the regression results also find that LET exerts the same most significant effect on college students’ CBBL as LM. The positive result of LET aligns with prior research on the influence of LET on learners’ learning persistence (Jung and Lee, 2018; Pursel et al., 2016). These studies suggest that LET shows a strong predictive power when learners engage in online learning and complete MOOCs. Therefore, college students who invest more time, emotions, and effort in the BL setting will be better able to utilize both online and offline resources, effectively planning and monitoring their learning progress, and can maintain their motivation to learn even when faced with challenges. Furthermore, SEF also has a positive impact on college students’ CBBL, which is in accordance with previous studies (Jiang and Liang, 2023). However, it is noteworthy that the influence of SEF is smaller than LM and LET, which is in contrast to the conclusions of some studies that emphasize the strongest influence of SEF in determining the actions taking to achieve their goals (Chen et al., 2022). The reason may be that LM (e.g., intrinsic interest, extrinsic rewards) serves as the direct driving force for behavior (Ryan and Deci, 2000), particularly in the BL environment, where college students must proactively adapt to the integration of online and offline modes. The intensity of motivation can directly determine college students’ continuance behavior toward BL. Moreover, LET plays a behavioral reinforcement role in BL; and high-frequency engagement immediately enhances college students’ sense of accomplishment, forming an action-feedback loop that directly promotes their continuance behavior (Jung and Lee, 2018). However, SEF influences college students’ confidence in completing learning tasks, and its effect is often moderated by specific task characteristics (Sun et al., 2019). In the BL contexts, even college students with high SEF, they may also experience weakened behavioral willingness if their practical experiences with the BL courses are negative, thereby limiting the direct impact of SEF on college students’ continuance behavior toward BL.

4. BCC significantly influences college students’ CBBL via the mediating effects of LM and LET rather than SEF.

Our results show that BCC can positively affect college students’ CBBL, and this effect is less than the impact of IST. Despite this, BCC exerted a positive and statistically significant influence on college students’ CBBL, this positive effect is in accordance with previous studies, which emphasized that BCC was positive related to students’ learning outcome and satisfaction (He et al., 2022; Purarjomandlangrudi and Chen, 2020). Moreover, in addition to the direct effect, BCC has an indirect influence on college students’ CBBL through LM and LET. This result can be supported by the social cognitive theory, which has suggested that learners’ behaviors would be strongly stimulated by environmental and external factors (Stajkovic and Luthans, 1998; Zhang et al., 2021). Exactly, the more distinctive the BL curriculum features, the greater the college students’ LM and LET, leading to a higher likelihood of continued learning in the BL environment. Therefore, when designing the BL courses, teachers should fully consider features such as learning flexibility, rich resources, diverse interactions, and personalized learning. The integration of these elements can enhance students’ learning experiences, increase their motivation and engagement, and finally effectively promote the development of continuance behavior of BL. Such designs not only meet the needs of different students but also stimulate their interest and initiative. Additionally, it is noteworthy that, SEF has no mediating effect in the relationship between BCC and college students’ CBBL. This result could be explained by that LM may play a more important role in this association between BCC and college students’ CBBL, replacing SEF as the mediator, and the influence of SEF may require time to accumulate, while the impact of LM is more immediate.

5. ITS can positively affect college students’ CBBL through the mediating role of LM, SEF, and LET.

The empirical results indicate that the level of ITS is a most predictive indictor on college students’ CBBL, which is consistent with prior research on the impact of perceived ITS on learning behaviors (Lee and Baird, 2021). As one of the most critical body in the BL environment, instructors play a key role in the designing, implementation, and assessment of the BL curriculum. Their teaching philosophies, goals, and methods directly influence the structure and content of the curriculum, while factors such as the resources provided, technical support, and interactions with students significantly impact the learning experiences and behavior of students. Thus, college students perceiving a strong ITS, such a clear guidance, timely and constructive feedback, encouragement, and easily acquisition of relevant resources, can be key factors for predicting their CBBL. Moreover, the positive relationship between ITS and college students’ CBBL is mediated by LM, SEF, and LET, that is, ITS can be used as an external stimulus to influence students’ internal psychological state, which in turn ultimately affects their learning behavior. This finding is line with extant studies clarifying that leaners’ LM and SEF were significantly influenced by teachers, interactions and other social factors (Jiang and Liang, 2023; Peng and Fu, 2021). Yang et al. (2023) investigated how SEF and motivation directly and indirectly affect the intention of beginners to persist in BL. Jung and Lee (2018) indicated that teaching presence and perceived usefulness would promote learning persistent through LET. Therefore, consisting with existing studies, ITS as a key stimulation, exerts a significant indirect effect on college students’ CBBL through LM, SEF, and LET. Namely, when students are at low level of LM, SEF, and LET, they are insufficient in independently learning abilities, which further need more supports and guidance from instructors in the BL environment.

### 6.2 Implications

BL, characterized by flexibility, interactivity, and a student-centered approach, effectively meets the growing demand for personalized learning among college students in the era of rapid “Internet + Education” development (Lv and Li, 2024; Zhao, 2022). This educational model significantly enhances learning outcomes by fostering autonomy and creativity. However, college students often encounter challenges in the BL environment, including high dropout rates, low persistence, and insufficient continuance behavior (Jiang and Liang, 2023; Jung and Lee, 2018), all of which undermine the effectiveness of BL education. Therefore, promoting sustained student engagement in BL to fully harness its advantages has become a critical issue. This study develops an integrated model that considers course characteristics, instructor support, and individual attributes, examining their impact on college students’ continuance behavior in BL courses. Based on the findings, this study offers policy recommendations from both learner and instructor perspectives to support the sustainable development of BL.

1. Cultivate college students’ personal attributes and enhance their enthusiasm, self-confidence and engagement in the BL environment. The empirical results suggest that college students’ personal attributes, including LM, SEF, and LET, have a significant and positive impact of their CBBL. Therefore, it is necessary to clarify how to cultivate students’ individual characteristics and stimulate their positive effects.

First, establish well-defined learning objectives. In the BL process, each course has a clear teaching objective. Based on this, college students also formulate a learning objective, so as to improve their sense of learning direction and purpose (Songsangyos et al., 2016; Yang et al., 2023). Furthermore, the overall objective should be divided into small stage goals according to the progress of the BL courses. These stage goals are specific, measurable and time-limited, which can be gradually achieved. By doing this, students will experience a sense of success and enhance self-confidence.

Second, promote interaction and cooperation. Undergraduates can enhance the social and engaging aspects of BL by participating in project-based collaborations, discussion groups, and other forms of interaction (Wei et al., 2022). At the same time, they can provide peer feedback and evaluation to help each other gain new perspectives and learning skills from their peers, filling in the gaps of BL approach.

Third, encourage reflection and self-evaluation. Students should reflect and record their BL progress to help them identify their strengths and weaknesses, so as to enhance their self-monitoring ability. Additionally, students can use self-assessment tools to regularly review their BL progress and identify problems in both online and offline learning. Meanwhile, they also need to effectively manage their time, especially during the fragmented offline time when various club activities, competitions and part-time jobs are dispersed (Zhou and Fang, 2024b), to improve their time utilization rate and offline learning effect.

Forth, improve LM and SEF with artificial intelligence (AI) tools. In the process of BL, college students should make full use of AI tools such as ChatGPT and DeepSeek, to construct knowledge graphs, integrate online and offline learning content, and leverage intelligent recommendations to accurately match learning resources. By doing this, they can dynamically adjust the learning content and progress and further improve learning effectiveness. In this process, AI not only enhances students’ LET but also helps them build SEF through the accumulation of successful learning experiences, ultimately enabling continuance learning behaviors in the BL environment.

2. Give full play the role of instructors in guiding and motivating BL to improve college students’ LM, SEF and sense of participation. Our findings indicate that ITS not only can positively affect college students’ CBBL, but also significantly promote college students’ CBBL through their LM, SEF, and LET. Therefore, the role of teachers’ supports in the BL environment cannot be overlooked (Feng et al., 2023; Lungu, 2013).

First of all, enhance digital teaching capabilities. Digital teaching level is the premise for instructors to provide help to students in BL. Instructors should be familiar with the use of various online interaction and management tools, such as LMS, tools related to online assessment and data analysis, to improve the effect of online teaching (Janse van Rensburg and Oguttu, 2022). In addition, instructors should master how to design more attractive and interactive BL courses that combine the advantages of online and offline to provide a rich teaching experience.

Second, increase interaction and provide emotional support. Instructors should actively engage with students in BL courses, which does not just answer questions, but also maintain contact with students through online discussions, feedback sessions, etc. to ensure that students can receive timely feedback after raising questions, thus enhancing their learning experience and sense of participation (Gutiérrez and Tomás, 2019; Wei et al., 2022). Additionally, instructors should use positive language and encouraging feedback to help students overcome challenges in BL and create an inclusive and stress-free learning environment (Zheng et al., 2024).

Third, provide online and offline guiding on a regular or irregular basis. Instructors should assess students’ BL progress regularly through online quizzes, tests, and assignments. After that, instructors should provide constructive feedback to help students identify weaknesses and make improvements (Feng et al., 2023). Meanwhile, instructors can engage in offline discussions with students to focus on learning difficulties and breakthrough points, guide them to reflect on their learning, and help find ways to improve (Rice et al., 2013).

Forth, effectively combine AI tools with teaching process. Teachers should leverage AI to recommend suitable BL resources according to students’ learning behaviors, course content, and instructional objectives. Meanwhile, AI-powered interactive tools such as ChatGPT and Socrative enable teachers to conduct intelligent Q&A sessions, real-time quizzes, and discussion analyses, thereby enhancing college students’ LET. Additionally, AI can serve as a teaching assistant by automatically grading assignments, reducing repetitive tasks, and allowing teachers to focus on BL and provide more personalized supports, which finally help enhance students’ SEF and CBBL.

3. Emphasize teaching design to fully reflect the characteristics of BL courses to enhance students’ motivation and engagement. From the empirical results, it can be found that BBC can positively and significantly affect college students’ CBBL, moreover, both LM and LET can significantly mediate the link between BCC and college students’ CBBL. Hence, it is crucial to design BL courses effectively to maximize their influence on college students’ CBBL (Yang and Pu, 2022).

First, increase interactivity. On one hand, it necessary to enhance student-teacher interaction through online discussions and real-time question-and-answer sessions. On the other hand, it also should promote student-to-student interaction by introducing group discussions, project collaborations and peer evaluations. Additionally, online learning communities also should be established to facilitate knowledge sharing, problem discussions, and experience exchanges. Meanwhile, invited industry experts can be invited to give online lectures to enhance the appeal of the BL courses.

Second, provide personalized learning paths. Course design should provide personalized learning content based on students’ learning progress, interests, and performance, helping them consolidate knowledge in a targeted manner. The course content can be divided into multiple modules, and students can choose the learning sequence and pace flexibly according to their own needs, thus enhancing their autonomy and flexibility in learning (Purarjomandlangrudi and Chen, 2020).

Third, organize diverse forms of learning contents. The BL course design should not only provide diverse learning materials such as text, video, and animation, but also include additional reading resources or external learning tools to help students deeply understand the BL course content (He et al., 2022). In addition, by combining theoretical knowledge with practical applications through case analysis and simulation practice projects, the practical value of the learning can be enhanced.

### 6.3 Limitations and future research

This study has several limitations that that can guide the focus of further research. First, this study adopts SOR framework and social cognitive theory as the theoretical foundation to explore the determinants of college students’ CBBL. In our study, BCC and IST are considered as the key external stimulus for college students’ psychological states and CBBL. Future studies could investigate other external stimulus. Especially in the context of the rapid development of artificial intelligence, it is worthy to explore how the characteristics of artificial intelligence, such as interactivity and personification, will affect the psychological states of college students and their continuance behavior in the BL environment. Second, this study uses questionnaires to collect cross-section data and the SEM to conduct the empirical analysis. Future scholars could use the scenario experiment method to compare the differences in college students’ CBBL in the experimental group and the control group, or collect panel data for quantitative analysis, so as to reduce the result bias caused by questionnaire data, as well as improve the stability and reliability of empirical results. Third, this study only focuses on college students who engage in BL on the Chinese university MOOC platform, and other online platforms are largely ignored. Future research can analyze and compare the differences concerning college students’ CBBL among different platforms, such as Wisdom Tree, Super Star Erya and Coursera, thereby improving the universality of conclusions.

### 6.4 Conclusion

According to social cognitive theory and the SOR framework, this study proposed a theoretical model of *“Contextual facilitators–Individual characteristics–Continuance behavior”* to investigate the relationships among external contextual factors (BCC and IST), individual factors (LM, SEF, and LET) and college students’ continuance behavior in the BL environment. Adopting SEM and AMOS software, 11 hypotheses have been examined in college students who have studied the BL courses on the Chinese MOOCs platform. The empirical results indicate that college students’ CBBL can be significantly promoted by BCC, ITS. LM, SEF, and LET. Specifically, BCC as an important stimulus, can significantly and positively affect college students’ LM, and LET, of which the impact on LM is the greatest, but has no significant on SEF; ITS can significantly stimulate college students’ LM, SEF, and LET, and the influence on LET is the strongest. Moreover, the impact of ITS on individual attributes is more than BCC. In turn, LM, SEF, and LET are the critical antecedents of college students’ CBBL, with significant and positive effects. Additionally, both LM and LET have mediating effects on the association between BCC and college students’ CBBL, but the mediating effect of SEF is insignificant, while the relationship between ITS college students’ CBBL is mediated by LM, SEF, and LET. These findings are beneficial for educators in Chinese universities who implement the BL model via the Chinese MOOCs platform, as they enhance students’ continuance behavior, which is crucial for both their learning outcomes and overall effectiveness.

## Data Availability

The original contributions presented in the study are included in the article/supplementary material, further inquiries can be directed to the corresponding author.
